# Factors Influencing Quality of Life for Disabled and Nondisabled Elderly Population: The Results of a Multiple Correspondence Analysis

**DOI:** 10.1155/2013/258274

**Published:** 2013-06-27

**Authors:** M. Avolio, S. Montagnoli, M. Marino, D. Basso, G. Furia, W. Ricciardi, A. G. de Belvis

**Affiliations:** ^1^Institute of Hygiene and Public Health, Università Cattolica del Sacro Cuore, Largo Francesco Vito 1, 00168 Rome, Italy; ^2^Dynamic and Clinical Psychology Department, Università “Sapienza”, Piazzale Aldo Moro 5, 00185 Rome, Italy

## Abstract

*Objectives*. The aim of our study is to examine the role of some factors (sociodemographic patterns, social relationship support, and trust in healthcare actors) on structure of quality of life among the Italian elderly population, by stratifying according to presence or absence of disability. *Methods*. Using data of the Italian National Institute of Statistics (ISTAT) survey, we obtained a sample of 25,183 Italian people aged 65+ years. Multiple Correspondence Analysis (MCA) was used to test such a relationship. *Results*. By applying the MCA between disabled and nondisabled elderly population, we identified three dimensions: “demographic structure and social contacts,” “social relationships,” “trust in the Italian National Health Services (INHS).” Furthermore, the difference in trust on the INHS and its actors was seen among disabled and non-disabled elderly population. *Conclusions*. Knowledge on the concept of quality of life and its application to the elderly population either with or without disability should make a difference in both people's life and policies and practices affecting life. New domains, such as information and trusting relationships both within and towards the care network's nodes, are likely to play an important role in this relationship.

## 1. Introduction

The 20th century has been characterized by a great advance in life expectancy; over the last century, chronic health problems have replaced infectious diseases as the dominant health care burden, and almost all chronic conditions are strongly related to aging. Only in the last few years many health care planners and governments have become aware of this phenomenon and population-based studies regarding age-related chronic diseases have been implemented. Despite the worldwide aging phenomenon, data regarding health and time trends referring to the health of the elderly population are still inadequate [1].

Welfare systems urge to address the social determinants and social gradients of health among the elderly population, for whom social relationships play an important role in access and use of higher quality healthcare services [2].

Among the elderly population, participation in social relationships is likely to be associated with better health status indicators [3–10]. Similarly, poor social relationships are likely to be associated with worse measures of quality of life [11, 12].

Furthermore, the association between social networks and health status is likely to be influenced by social context and therefore by behavioral, cultural, psychological, and physiological condition and material instability [13, 14].

Over the last years, in health and social science fields, growing interest has been devoted to services, programs, and treatments that improve individual quality of life. For this reason perceived well-being of service users is crucial to assess the effects and importance of treatments and services and determining quality of life dimensions. The concept of quality of life for disabled people has different meaning and the improvement of life conditions becomes a shared goal of many programs aimed at these people, acquiring great relevance in outcome analyses.

For this reason, determining and promoting the quality of life of consumers of educational, social, health, and/or healthcare services become a priority [15]. 

A recent analysis of the literature by Schalock [16] on disabled people about quality of life domains yielded several indicators. The vast majority of these indicators were related to seven core quality of life domains: interpersonal relations, social inclusion, personal development, physical well-being, self-determination, material well-being, and rights.

Following our previous studies [17], we realized that the application of one-dimensional measures in social relationships and the limits of the application of multiple logistic regression models were not exhaustive to fully explore the influence of other linked dimensions (e.g., trust is a basic element in healthcare as well as social care and it is at the same time a difficult phenomenon to conceptualize) and their relationships to quality of life among the elderly population.

The aim of our study is to explore—by stratifying the subjects into disabled and nondisabled elderly population—the influence of the following factors on structure of quality of life: “interpersonal relations,” “social inclusion,” “physical well-being,” “self-determination,” “material well-being,” and “personal development.”

## 2. Materials and Methods

The study was conducted using data from the last available version of National Survey on “Health conditions and health care services use,” a five-year nationwide survey conducted by the Italian National Centre for Statistics (ISTAT) [18]. We focused on a sample of 25,183 elderly population (aged 65+) residing in Italy between 2004 and 2005.

The sample was stratified by the presence or absence of disability:

- 2,887 disabled people;
- 22,296 non-disabled people.

We assumed that people with disabilities would perceive their health status and quality of life differently than people without disabilities [19]. ISTAT, according to International Classification of Impairments, Disabilities and Handicaps (WHO 1980), defines disability as impairments, activity limitations, and participation restrictions. “Health conditions and health care services use” survey shows that disability population is 2,6 million, about 4,8% of population older than 6 years old. Data on disabled and non-disabled people were categorized according to the ISTAT classifications [18]. The indicator for disability was built up by ISTAT making refer to Organization for Economic Cooperation and Development (OECD) set of questions about International Classification of Impairments Disabilities and Handicaps (ICIDH) of World Health Organization (WHO) to study specific disability dimensions: physical disability (confinement-troubles in walking, lower yourself, going up/going down, and brush), people care (functional autonomy), and dimension of communication (sight, hearing, and speech). 

Statistical weight coefficients were assigned to the data by the carrying rate of the sample size.

According to findings of Schalock [16], the collected data from the survey questions dealing with the social determinants of health were categorized into “interpersonal relations” (interactions, relationships, support-emotional, physical, financial, and feedback), “social inclusion” (community integration and participation, community roles, social support network, and services), “physical well-being” (health, activities of daily living, leisure, and access to health care), “self-determination” (autonomy/personal control, goals and personal values, choices-opportunities, options, and preferences), “material well-being” (financial status, employment, and housing), and “personal development” (education, personal competence, and performance) (Table 1). By considering all Schalock dimensions, all mentioned variables were included in the MCA analyses.

### 2.1. Statistical Analysis

A preliminary descriptive analysis was carried out to address the modalities of each variable in the same direction, so as to let them occur together. To explore the factors influencing the perceived quality of life among the elderly population, we applied the Multiple Correspondence Analysis (MCA).

MCA is a descriptive/exploratory technique designed to analyze simple two-way and multiway tables containing some measures of correspondence between the rows and columns.

MCA is used to analyze a set of observations described by a set of nominal variables. This is a particular/special technique of Factor Analysis [20] that has been chosen for flexibility and applicability. The results provide information which is similar in nature to those produced by Factor Analysis techniques, allowing to explore the structure of categorical variables included a table. The interpretation of the axes is based upon the contributions of the categories.

The explained inertia (i.e., variance) is therefore severely underestimated, and we used the correct formula that provides a better estimate of the inertia, extracted by each eigenvalue. The correct formula is provided by Benzécri [21]. The interpretation in MCA is often based upon proximities between points in a low-dimensional map (i.e., two or three dimensions). As well as for Correspondence Analysis (CA), proximities are meaningful only between points from the same set (i.e., rows with rows, columns with columns). Since the interpretation of MCA is more delicate than simple CA, several approaches have been suggested to offer the simplicity of interpretation of CA for indicator matrices. When the indicators were a very low frequency (<2%), we randomly (re)assigned this variables, by using SPAD software, to control so-called “rare statistic modality.”

By applying MCA, variable numbers were reduced in the latent factors. On each of the factorial axes, we obtained a discrimination measure to represent the intensity with which the variable explained the axis [21]. Moreover, we analyzed the relative contributions of variables and we assessed which modalities are represented on the axes. Each MCA dimension's name was arbitrarily attributed according to the interpretation of its list of variables.

All analyses were performed using SPSS (Version 17) and SPAD (Version 5).

## 3. Results

The disabled sample shows that 43.40% of disabled people are married if compared with non-disabled (58.60%) and they live alone more frequently (32.21% versus 27.12%). Above one fifth of the elderly population, with presence or absence of disability, declares to live too far from own relatives' home. “In case of life troubles,” 81.29% of the disabled aged 65–74 and 83.86% aged 75 and more can instead count on their relatives; these percentages for non-disabled rise to 83.74% and 87.33%, respectively. Disabled people declared to need homecare services for the 33.91% and home assistance assigned by Local Health Unit (LHU) for the 18.08%; among non-disabled people these percentages decrease to 5.32% and 2.03%, respectively.

In addition, descriptive analysis shows that in the last year, 53.80% of overall sample perceived quality of National Health Service as “the same” or “better.” In order to take decision on their own health, more than 87.01% of disabled people used to ask an advice to the health professional if compared to 85.58% in non-disabled sample.

By applying the MCA among the disabled elderly population, we identified three dimensions (axes), which explained a 71.64% improved estimate of the inertia among the ten factors. For the first factorial axis (“demographic structure and social contacts”), the principal discrimination measures are included in “interpersonal relation” and “social inclusion” configured in living alone, marital status and availability, and mobile for one's own relatives. For the second axis (“social relationships”), the discrimination measures can be mostly associated with “interpersonal relations” (in case of life troubles, my family can trust/count on: friends, neighbors, non-profit associations). The third factorial axis (“trust in the INHS”) was made via measures related to trust in the General Practitioner (GP) or specialist. The percentages of total variance explained by each dimension are the following: dimension 1 explained 34.69% of the total variance while dimensions 2 and 3 explained 20.84% and 16.12%, respectively (Table 2).

In the non-disabled sample, we identified three main dimensions which explained 77.38% of the improved estimate of the inertia among the ten factors. The percentages of the variance explained by each dimension are the following: dimension 1 that explained 40.20% of the total variance, and dimensions 2 and 3 that explained 21.44% and 15.74%, respectively. Among the non-disabled elderly population in the first factorial axis (“demographic structure and social contacts”), the principal discrimination measures are associated with “interpersonal relation” and “social inclusion,” these including living alone, marital status, and a mobile phone not available for own relatives. The second axis (“social relationships”) includes interpersonal relations and the availability of support and advice (“In case of life troubles, my family can trust/count on: friends, neighbors, non-profit associations”). Finally, in the third factorial axis (“trust in the INHS”), there is a relevant influence of “trust on GP” and “trust on specialist”, which are classified as “self-determination” according to Schalock's work [16] (Table 3).

## 4. Discussion

In the last years there was an increasing interest in the social and psychological dynamics of the perceived status of well-being, including factors related to social relationships/support, interpersonal trust, internal control, autonomy/independence, self-confidence, aspirations/expectations, and values having to do with family, job, and life in general [16]. 

Social relationship affiliations and social activity participation could be influenced by the disability [14, 17], and cross-sectional surveys do not provide assistance in the investigation of the strength and direction of such a relationship. Furthermore, being disabled can lead to unfavorable outcomes in both the access and quality of healthcare services, as well as outcomes regarding expectations and trust on its actors [20, 22].

Previous studies explored the influence on self-perceived quality of life of the health status, the social relationships/social inclusion, and the access to healthcare services.

Our study adds new findings on the role of sociodemographic patterns, social relationship support, and trust to healthcare actors on quality of life, among the Italian elderly population by stratifying the sample according to the presence or absence of disability.

In addition, the application of MCA helped to better test the relationship between quality of life and social/health factors in the elderly population.

The MCA analysis confirmed the role of social relationships on the quality of life (first dimension: “structural socio-demographic conditions”). Its role was the most influent, more among disabled than in non-disabled elderly population, respectively, thus confirming previous analysis [7, 10]. Within such dimension, the factor “marital status” is oppositely shaped among the two strata, thus confirming, among the elderly population, not such a positive perception of being married on quality of life (e.g., among women in Italy) [17].

Elderly and disabled people can count on the supportive, active role of their own spouse and of family as a whole, and they are likely to recognize family integration as relevant for the individual inclusion in the community. Such a network would count on availability; that is why disabled people are more likely to recognize the utility of a permanent connection with their own relatives (e.g., by mobile).

Among the disabled elderly population, to count on elective social relationships (i.e., “counting on friends” and “counting on neighbors”) is more developed if compared to the overall elderly population. This would be due to the necessity of a supportive network against isolation, exclusion, and other additional negative life occurrences [23]. In addition, among the stratum of the disabled in our sample, a bigger role of voluntary associations in supporting and counseling the disabled on health issues is recorded. Such a role was confirmed by its interaction with structural socio-demographic conditions as well [24]. 

These findings would be particularly useful in the design of welfare policies towards the disabled elderly population [25]. In particular, the incoming financial constraints are urging the welfare agencies to address the main determinants for social inclusion for the elderly people, and such findings would help them to target the most effective (and cost-effective too) policies and to stratify among disable and not disabled. 

Previous studies assessed that access and utilization of social services are also influenced by features of the caregiver, socioeconomic factors, and the available resources. While caregivers' needs influence the services use, the family enabling factors are the most important predictors of the amount of services used [26].

Among the disabled enrolled in the survey, trust in INHS involves mostly GPs, thus confirming that a daily consolidated relationship regarding health issues is likely to be privileged.

As for the non-disabled, the analysis confirmed a protective role of “counting on neighbors” and “counting on friends” on the quality of life. “Counting on people belonging to voluntary associations” is likely to play a relevant role as well.

Furthermore, a role of trust on INHS and its actors emerged, even though with different relative attributes to GP and specialist in the disabled and non-disabled strata, respectively. 

In countries like Italy with a socialized health care system, trust might be of two types [27, 28]. The first type is the trust in doctors and nurses we see in GP clinics and hospitals, together with the unspoken trust in all of the unseen support staff in the laboratories and offices. The second type is the trust in systems of the INHS to deliver the health care that people need, at least most of the time.

Our analysis revealed different figures regarding trust in INHS' actors; trust in specialist, rather than in the GP, confirms different attitudes and expectations regarding the health delivery system among the non-disabled, whose satisfaction and trust seems to derive from a more selected demand of specialized services [29]. 

It should be stated that our analysis contains certain limitations. One limitation derives from our cross-sectional design, which means that temporal directions of associations between reciprocally connected variables could not be defined.

The entire social relationship dimension was not completely explored in the ISTAT questionnaire. A low power of analysis inside the kin or nonkin networks was a limit of such an investigation [30].

Trust regarding care would take into account healthcare as well social care.

As clear questions regarding social supports/services were contained in the multipurpose survey, the social services were found to be inadequately supplied to the disabled, and public financial help to their families was also seen to be inadequate according to our analysis [31].

Unfortunately, the multipurpose survey [18] does not explain which kind of interventions is provided by voluntary associations, so as to disaggregate between the disabled and the non-disabled elderly population.

The comprehensive interactive role of information and trust in relationships and self-perceived health in the elderly population has not been widely investigated, due to the scarcity of information in the ISTAT questionnaire.

A limit of the MCA involves its mainly explorative role [21]. Further analysis is needed to evaluate the role of the key results.

By applying MCA, together with marital status (“unmarried” or “not yet married”) and “living alone,” we found out that the most outstanding dimensions in the relationship with quality of life among the elderly population were the use of healthcare services, the trust on own doctors (GP and Specialist), and the availability of a confidant/adviser on health problems.

Knowledge regarding the concept of quality of life and its application to the elderly population either with or without a disability should make a difference in both people's lives and the policies and practices that impact those lives [16].

Social relationships represent an important factor in improving quality of life among the elderly population, and new domains are likely to play an important role in this relationship.

## Figures and Tables

**Table 1:** A framework of determinants on quality of life.

Dimensions	Variables (modalities)
Interpersonal relations (interactions, relationships, support-emotional, physical, financial, feedback)	Living alone (no, yes)
	Marital status (married, unmarried, or not yet married)
	In case of life troubles, my family trust/count on: relatives, friends, neighbors, nonprofit associations, other? (no, yes)
	Home health/social career on behalf of the municipality (no, yes)
	Home worker (no, yes)
	Elderly/handicapped care (no, yes)

Social inclusion (community integration and participation, community roles, social support network, services)	Distance too long between own home and relatives' home (no, yes)
	Do your relatives use a mobile? (yes, no)
	Do you have telephone at home? (yes, no)

Physical well-being (health, activities of daily living, leisure, access to health care)	Physical disability (no, yes)
	Mental disability (no, yes)
	Need to home care services (no, yes)
	Recourse to health-rehabilitation services in the last three months (no, yes)
	Home health career on behalf of local health unit, (no, yes)
	Do you ask someone for important decision on own health? (I ask my GP, I ask a specialist, I ask my private physician, I ask other health professionals, I take final decision by myself)
	Flu vaccination in the last twelve months (yes, no)
	Frequency of blood hypertension check (At least once a year, less than once a year, never)

**Table 2:** Factor sets of the three main dimensions among the disabled elderly population.

Dimensions	Dimension 1: Demographic structure and social contacts (relative contribution)	Dimension 2: Social relationships (relative contribution)	Dimension 3: Trust in the INHS (relative contribution)

Inertia	34.69%	20.84%	16.12%

Factors	Marital status (unmarried or not yet married = 9.6, married = 13)	My family count on friends (no = 7.4, yes = 11.6)	Trust in GP (no = 14.3, yes = 5.9)
	Living alone (yes = 19.5, no = 9.7)	My family count on neighbors (no = 7.3, yes = 9.9)	Trust in specialist (no = 3.5, yes = 11.0)
	Availability of mobile for own relatives (yes = 6.4, no = 6.6)	My family count on people belonging to voluntary association (no = 2.3, yes = 14.6)	

**Table 3:** Factor sets of the three main dimensions among the non-disabled elderly population.

Dimensions	Dimension 1: Demographic structure and social contacts (relative contribution)	Dimension 2: Social relationships (relative contribution)	Dimension 3: Trust in the INHS (relative contribution)
Inertia	40.20%	21.44%	15.74%

Factors	Marital status (unmarried or not yet married = 14.3, married = 10.0)	My family count on friends (no = 14.8, yes = 15.1)	Trust in GP (no = 18.4, yes = 6.3)
	Living alone (yes = 19.8, no = 7.3)	My family count on neighbors (no = 12.7, yes = 13.2)	Trust in specialist (no = 4.0, yes = 13.3)
	Availability of mobile for own relatives (yes = 5.4, no = 9.5)	My family count on people belonging to voluntary association (no = 1.7, yes = 13.1)	
